# Will an International Qualitative Study of Crohn’s Disease Patient Experiences and Preferences Evolve the Future of Disease Monitoring?

**DOI:** 10.1093/crocol/otad013

**Published:** 2023-02-25

**Authors:** Rocio Castrillon

**Affiliations:** Crohn’s & Colitis Foundation, New York, New York, USA

Crohn’s disease patients undergo a significant amount of testing and monitoring through their disease course. While clinicians focus their primary efforts in treating Crohn’s disease patients with therapeutic options, the actual patient experience becomes secondary in disease monitoring. Though patient preferences may be taken into consideration, there does not seem to be a significant amount of time spent discussing what the patient is experiencing—physically, emotionally, mentally. Given the complex nature of living with Crohn’s disease, it was reassuring to see the following study be prioritized in the inflammatory bowel disease (IBD) world.

“Crohn’s Disease Patient Experiences and Preferences with Disease Monitoring: An International Qualitative Study” utilized group interviews with adult patients to better understand patient experiences and preferences as it relates to monitoring their Crohn’s disease. The results of the study have been made available to guide and inform disease monitoring strategies, improve patient engagement and optimize care to ultimately have a more patient-centered approach. But the ultimate question remains…will clinicians truly utilize the findings of this study to incorporate into their practices and with their patients?

With endoscopic healing being considered the gold standard in clinical practice and a target for clinicians and patients alike, the necessary procedure to determine endoscopic healing—colonoscopy—is invasive, uncomfortable, and physically and mentally draining for many IBD patients. Over the course of IBD patients’ disease journeys, this procedure, along with many others, are repeated numerous times. As can be assumed, most patients prefer less invasive procedures.^1^ Unfortunately, those options are limited and may not be able to provide strong guidance for clinicians. Additionally, some of the additional testing options, such as MRE (magnetic resonance enterography), CTE (computed tomography with enterography), and IUS (intestinal ultrasound), have limitations themselves, including: limited availability (particularly with IUS), high out of pocket costs (MRE), and repeated exposure to radiation (CTE).^2–4^

Despite the importance of patient involvement in the decision-making process of IBD journeys, there has previously been little data supporting the actual patient population playing an active role. This study, however, was able to not only focus on the patient population, but also gauge their experiences and preferences and ultimately increase patient engagement. Focusing a study entirely on patient preferences is pivotal in enabling change in the treatment of Crohn’s disease (and ulcerative colitis) patients alike.

There were 3 main themes that were prioritized within this study which included: patient preferences for monitoring, emotional responses of monitoring and improving disease monitoring. First, the patient preferences for monitoring focused primarily on the least invasive options, with patients understanding the limitations that those options bring. Patients ultimately accepted that the most invasive testing options would yield the best results for both clinician and patient, therefore they understood that when there was a need for these procedures, they would accept it. Disease monitoring was contingent upon disease activity for most of the patients in the study and it was noted that those who were in remission were monitored less, whereas those with high disease activity accepted the reality of needing more close monitoring. One of the key highlights to note is the preferred method of monitoring is IUS as it provides accurate results without the invasiveness of other procedures, but the greatest challenge is availability of IUS globally. IUS has a huge potential for growth given the patient preference, the accuracy of the results and therefore the hope is that it can and will be readily available for clinicians everywhere to use. If there is a main takeaway from this study, it is that IUS becomes a standard measure of care for disease monitoring.

In addition to the logistics of disease monitoring considerations, there is a need to understand how important and impactful the emotional responses to monitoring are. Given the many varied emotions patients have with living with IBD, depending on their disease state, duration of disease and even extenuating factors unrelated to their Crohn’s disease, this is an opportunity for clinicians to truly understand their patients. While close monitoring instinctively causes worry and anxiety amongst all patients, it was found that some patients also created a needed space between themselves and their disease, almost as a way to separate their disease from their life—an understandable need.

Separately, there are many sociodemographic variables to be considered in disease monitoring, such as: employment status, occupation, housing, insurance, age, sexual orientation, race, and ethnicity. Patients also focused on recommendations for ways to improve disease monitoring with IUS once again coming up as the greatest priority. The high-information yield that IUS offers is what makes patients eager to learn more about this monitoring tool and have it utilized as baseline monitoring.

Along with the patient-centric discussion about disease monitoring, patients expressed a need for communication and knowledge sharing to help make informed decisions. One valuable suggestion was the development an app which could provide real-time access to data and clinical interpretations of the data. Additional suggestions included visualization or charting of results which would allow for a patient to “see” what the physician sees and better understand their own medical situation. Although patients tend to gauge disease state on symptoms they are feeling, they believe gaining a better understanding of what clinicians see will help them understand and agree to changes in treatment, if needed. Self-administered testing was requested by patients as they felt it would allow for self-monitoring of their disease activity and potentially expedite care. If they could test at home, they’d be able to see real-time results and be able to act upon it much more quickly than waiting for prior authorizations, scheduling, insurance, etc.

Finally, there was a desire for shared decision making, trust, and recognition of patients as experts. Patients ultimately feel that the best way to monitor disease is to be able to have ongoing communication with their medical team, being able to make decisions along with their clinician, and ultimately gaining the trust and confidence of their physician accordingly so that patients are treated as equals in decision making. Most IBD patients want to be empowered in their disease journey and will advocate for themselves. But the acceptance of this mindset needs to be accepted by clinicians for this disease monitoring process to be successful. Patients should be at the forefront of all considerations as it relates to disease management and monitoring and while this study brings strong relevance for patients along with clinicians, the hope is that the findings are truly utilized for change in the management and care of Crohn’s disease patients.
